# Effect of Silver Nanoparticles on the Optical Properties of Double Line Waveguides Written by fs Laser in Nd^3+^-Doped GeO_2_-PbO Glasses

**DOI:** 10.3390/nano13040743

**Published:** 2023-02-16

**Authors:** Camila Dias da Silva Bordon, Jessica Dipold, Niklaus U. Wetter, Wagner de Rossi, Anderson Z. Freitas, Luciana R. P. Kassab

**Affiliations:** 1 Departamento de Engenharia de Sistemas Eletrônicos, Escola Politécnica da Universidade de São Paulo, Av. Prof. Luciano Gualberto, 158, Travessa 3, São Paulo 05508-900, SP, Brazil; 2 Instituto de Pesquisas Energéticas e Nucleares, IPEN-CNEN, 2242, Av. Prof. Lineu Prestes, São Paulo 05508-000, SP, Brazil; 3 Faculdade de Tecnologia de São Paulo, CEETEPS, Praça Cel. Fernando Prestes, 30, São Paulo 01124-060, SP, Brazil

**Keywords:** germanate glass, fs laser processing, double line waveguide, active optical component, Ag nanoparticles

## Abstract

Nd^3+^-doped GeO_2_-PbO glass with silver (Ag) nanoparticles (NPs) are produced with double line waveguides through fs laser processing for photonic applications. A Ti:sapphire fs laser at 800 nm was used to write the waveguides directly into the glass 0.7 mm beneath the surface. This platform is based on pairs of parallel lines with spacing of 10 µm, each pair being formed by two identical written lines but in two different configurations of 4 or 8 separately processed lines, which are coincident. The results of optical microscopy, absorbance measurements, refractive index change, beam quality factor (at 632 and 1064 nm), photoluminescence, propagation losses, and relative gain at 1064 nm are presented. The structural changes in the glass due to the presence of Ag NPs were investigated by Raman spectroscopy. At 632 and 1064 nm, x,y-symmetrical guiding was observed, and for both kinds of overlapping pulses, a refractive index alteration of 10^−3^ was found in both directions. Photoluminescence growth of ~47% at 1064 nm was observed due to the plasmonic effect of Ag NPs. In dual waveguides containing Ag NPs, the relative gain obtained increased by 40% and 30% for four and eight overlapping lines, respectively, at 600 mW of 808 nm pump power, when compared to waveguides without those metallic NPs. We highlight the resultant positive internal gains of 5.11 and 7.12 dB/cm that showed a growth of ~40% and ~30%, respectively, with respect to the samples without Ag NPs. The increase in photoluminescence and relative gain were related to the local field growth produced by Ag NPs. The present results show that the addition of Ag NPs impacts positively on the optical performance at 1064 nm of double line waveguides processed by fs laser writing in Nd^3+^-doped GeO_2_-PbO glass, opening news perspectives for photonics.

## 1. Introduction

The fs laser processing system has been largely used and competes with clean room processing, which requires more complex procedures and higher costs. Two types of basic waveguides can be obtained using the fs laser processing method: single or double-line. In the first type, the structural modification of the material induced by the laser causes a positive increase of the refractive index, and light confinement takes place within the laser written trace [1], whereas a negative refractive index change occurs in the second type of laser written trace. In this last case, guiding cannot occur within the trace itself, but the light is guided in the region between two or more written traces or lines. Several glassy systems [2,3,4] operate with single-line waveguides. On the other hand, dual line waveguides represent another technique that has been demonstrated in a number of hosts, including glass and crystals [5]. We reported the first results of dual waveguides written by fs laser in undoped TeO_2_ and GeO_3_ glass [6]. Then, using the same architecture, active dual waveguides in Er^3+^/Yb^3+^-doped GeO_2_-PbO glass were reported by direct fs laser processing. In this case, a relative gain of 7.5 dB/cm for 105 mW of pump power at 980 nm and a positive internal gain of 4.6 dB/cm were achieved [7]. These results demonstrated that Er^3+^/Yb^3+^-doped GeO_2_-PbO glass are promising materials for the fabrication of integrated amplifiers. More recently, we reported a different platform for the dual waveguide configuration [8] with several collinear overlapping lines. In this case, the same focusing condition and energy were used, but a higher laser beam scanning speed (0.5 mm/s) was selected when compared to the previous work (Er^3+^/Yb^3+^-doped GeO_2_-PbO) [7] in which a much slower speed was used (0.2 mm/s). For the present work it was necessary to use the new method [8], as neodymium absorption at 800 nm is in resonance with the fs laser’s emitting wavelength (also 800 nm), causing fractures at low writing speeds in the glass due to the accumulation of heat in the processing region. In order to avoid low writing speeds but still achieve a high density of laser pulses along each written trace, which is necessary for obtaining a strong change in index of refraction, a method was developed consisting of writing each line at a much higher speed (small density of pulses) but then retracing it several times after a suitable time interval (40 s), necessary for cooling the processed region. With this new method, two parallel lines form a dual waveguide, where each line is produced by the superposition of four or eight coinciding lines. Positive internal gains of 3.61 and 5.56 dB/cm at 1064 nm were obtained for four and eight superimposed lines, respectively, demonstrating [8] that this current double line structure for Nd^3+^-doped GeO_2_-PbO glass contributes to promising applications in photonics, such as manufacturing lasers, integrated amplifiers, and lossless components. These results motivated the present investigation that uses the aforementioned dual line methodology in Nd^3+^-doped GeO_2_-PbO glass with silver (Ag) nanoparticles (NPs). This host doped with rare-earth ions and metallic NPs has been extensively studied and demonstrated many photonic applications that also motivate the present work. Rare-earth ion-doped glass based on GeO_2_ demonstrated photoluminescence enhancement due to localized plasmon effects of Ag-NPs [9,10,11,12]. Glass based on GeO_2_ were shown to be efficient materials for photovoltaic devices [13,14] and also for white light generation and tunable visible light emission devices [15,16]. 

Motivated by previous reports about the intense local field that appears around the Ag NPs and contributing to the enhanced photoluminescence of the rare-earth-doped GeO_2_-PbO glass [11,12,15], in this study, we present for the first time the influence of these metallic NPs on the optical properties of double line waveguides produced by fs laser processing in Nd^3+^-doped glass with the same composition (GeO_2_-PbO) as the aforementioned references. The effects on the structural changes are observed through Raman spectroscopy; passive (refractive index change, M^2^ beam quality factor at 632 and 1064 nm, and propagation losses) and active (relative gain at 1064 nm) characterizations are exposed. 

## 2. Materials and Methods

### 2.1. Glass Preparation and Waveguide Fabrication

The glass was prepared using the composition (in wt%) 40GeO_2_-60PbO; then, 1.0 wt% of Nd_2_O_3_ and 2.0 wt% of AgNO_3_ were added. The results of the samples without Ag NPs were also used for reference [8]. The samples were prepared with the melt-quenching technique using a high purity alumina crucible (99.999%), 1200 °C melting temperature, and a glass rod to homogenize them; the velocity of the rod had to be chosen carefully in order not to compromise the optical quality. Then, the melt was poured into preheated brass molds, and annealing at 420 °C (for 1 h) was conducted. This procedure is relevant to reduce internal stress and to make the samples less fragile. After that, cooling to room temperature was performed inside the furnace, and then the samples were finally polished. Samples 2 mm in thickness and with parallel faces were prepared for the experiments. Additional annealing was carried out to obtain the nucleation of metallic NPs by thermal reduction following the procedure used in previous studies [11,12,16,17]. 

**Figure 1:**
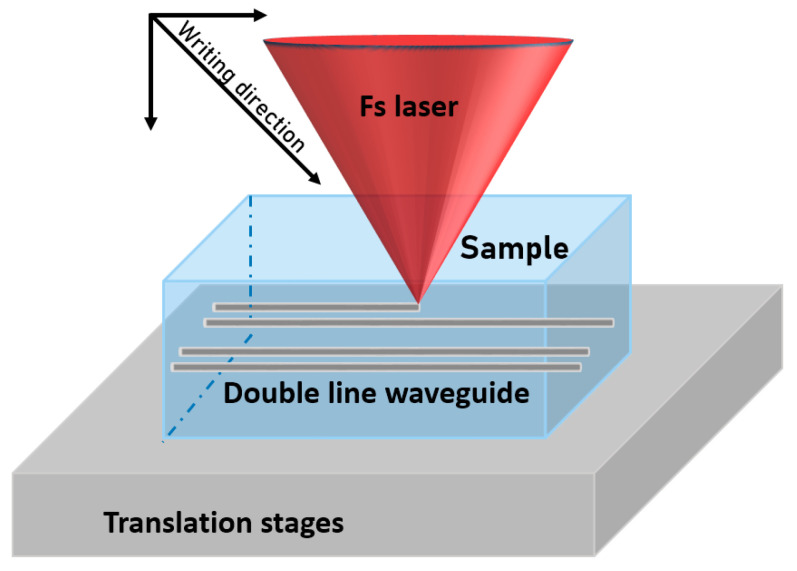
Set-up used for double waveguide processing.

The waveguide processing parameters reported in our previous work were used in the present work [8]. Figure 1 illustrates the configuration used for the waveguide processing performed at 800 nm by a focused femtosecond (fs) Ti:sapphire laser with a temporal pulse length of 30 fs. The beam was focused 0.7 mm below the surface (uncorrected due to the index of refraction) using a lens with a focal length of f = 20 mm and a diameter at the focus, in the air, calculated at 3.4 μm. A pair of parallel lines was written with a spacing of 10 µm, each line being composed of 4 or 8 coincident lines, using the parameters presented in Table 1. For the absorption measurements, a sample was prepared with a large number of parallel lines, spaced at 10 μm, making up an area of irradiated glass of 2 × 2 mm^2^ in order to produce as many Ag NPs as possible within this area.

It is worth noting here that the real position and number of overlapping pulses inside the glass are very difficult to calculate due to the autofocusing effect of the laser pulse close to the focal region. While the passage of the laser beam through the air-to-glass interface, with a refractive index close to 2 [18], increases the distance from the focal point to the surface and also its diameter, the nonlinear effect of autofocusing decreases this distance, and also the diameter of the focal point, spreading the absorbed pulse energy in a volume of hundreds of microns above and below this new focal point [19,20]. In this way, the written line, where the change in refractive index occurs, is no longer “one-dimensional”, but it looks more like a three-dimensional wall with a height much greater than its width, as will be shown.

Taking these conditions into account, the energy and numerical aperture of the lens used were determined in previous works as 30 μJ and 0.23, respectively. Choosing a low numerical aperture decreases autofocusing effects and increases the length of the refractive index modification “line”. Thus, there is a lower chance of physical damage to the material and also a larger focus region with a consequent increase in the guidance region in the collinear direction to the incident laser beam. By scanning this extended guiding region with the beam mode, it is possible to select the region of highest transparency. With this larger guiding region, a lower Fresnel loss is expected, and the portion with the best optical quality of the material can still be chosen. Hence, the energy used was fixed at a safe value so as not to cause damage; all the conditions of focus, speed, and repetition rate are shown in Table 1.

### 2.2. Characterization

Raman spectroscopy and optical microscopy measurements were performed in the waveguide region using a LabRam HR Evolution—HORIBA and a Leica DMLP polarizing light microscope with a MC170 HD camera, respectively. 

By measuring the *N.A.* (numerical aperture) of the waveguide, it is possible to determine the refractive index as follows [21]:

(1) $$N.A.=\sqrt{n_{1}^{2}-n_{2}^{2}}\approx \sqrt{2n_{2}\Delta n}$$

**Figure 2:**
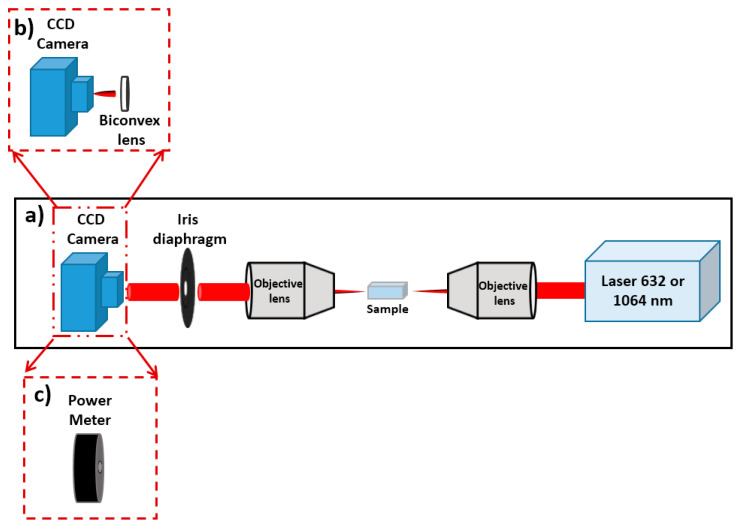
Experimental setup used to determine (**a**) the mode images and the numerical aperture, (**b**) the beam quality (with flat-convex lens), and (**c**) the propagation losses.

In the equation above, *n*_1_ and *n*_2_ represent the refractive index of the unaffected region between lines and that of the written lines, respectively. Figure 2a shows the arrangement used to obtain the mode images and the refractive index change at 632 nm; to determine the beam quality, an additional biconvex lens was added, as shown in Figure 2b. The focal distance of this biconvex lens was 35 mm. 

By using the equation below [22], the value of *M*^2^ was determined at 1064 nm:

(2) $$M^{2}=\frac{\theta_{ideal}.w_{0ideal}.\pi }{\lambda }\;$$

where *θ_ideal_* represents the half-angle beam divergence (obtained from *M*^2^) and *w*_0*ideal*_ (the gaussian beam waist radius), both measured at 632 nm (Figure 2b).

The cut back method was used to determine the propagation losses at 1064 nm [8] with a similar experimental setup as in Figure 2a, replacing the CCD camera with the power meter (Figure 2c) and using Equation (3) below, where *P_1_* and *P_2_* are the powers related to different lengths (*d_1_* and *d_2_*) of the sample.

(3) α [dBcm]=−10logP2P1d2−d1

The photoluminescence was determined by exciting the samples with a continuous-wave diode laser operating at 808 nm. Then, the signal was collected in the perpendicular direction to the incident beam and analyzed by a monochromator equipped with a Ge (germanium) photodetector with output coupled to a lock-in amplifier connected to a computer.

Absorption measurements were carried out with a UV-VIS-NIR Spectrophotometer—Cary 5000, operating from 350 to 1000 nm, to verify the incorporation of rare-earth ions and the presence of the plasmonic resonance band from the Ag NPs. The measurement was performed in two different regions of the sample specially prepared for this measurement, as mentioned before: in an irradiated area of (2 × 2) mm^2^ of the glass with many lines, which were separated by 10 µm and composed of 4 coincident overlays; and at the opposite end of the sample (with no fs laser irradiation). 

The relative gain (signal enhancement) at 1064 nm was determined using the previous procedure already reported [8], with probe laser operating at 1064 nm (400 nW of power) and pumping laser operating at 808 nm (600 mW of maximum power).

Equation (4) [7,8,23] was used to calculate the relative gain (*G*), where *P_signal_* and *P_ASE_* represent the signal power measured at the sample output (with the signal laser turned on and the pump laser turned off) and at the amplified spontaneous emission (*ASE*) with just the pumping laser turned on, respectively. When the pumping and probe lasers were switched on, *P_signal+ASE_* was obtained. The signal power of the stimulated emission (SE) at 1064 nm refers to *P_signal+ASE_* − *P_ASE_*, and *d* represents the sample length (0.5 cm).

(4) $$G\left[\frac{db}{cm}\right]=\frac{10\times log\left(\left(P_{signal+ASE}-P_{ASE}\right)/\;P_{signal}\right)}{d}$$

## 3. Results and Discussion

### 3.1. Optical Results

**Figure 3:**
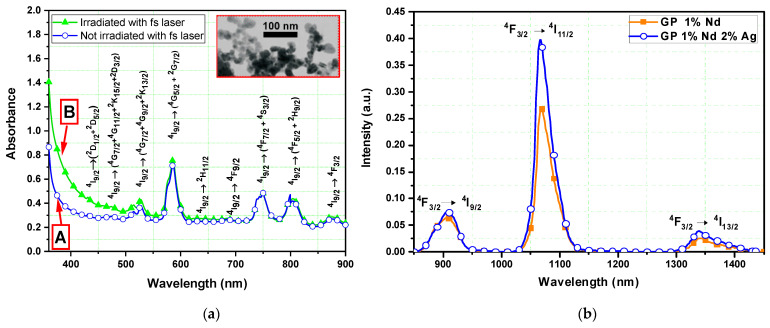
(**a**) Absorbance results of GeO_2_-PbO glass doped with Nd^3+^ and Ag NPs in two different regions of the sample (in laser focal region); the inset shows the TEM image of Ag NPs [11]; (**b**) photoluminescence spectra for Nd^3+^-doped Ge_2_O-PbO glass without (square) and with (open circles) Ag NPs.

The absorption bands associated with the Nd^3+^ transitions starting from the ground state, ^4^I_9/2_, are presented in the absorption spectra of Figure 3a for the GeO_2_-PbO samples doped with 1.0 wt% of Nd_2_O_3_ and 2.0 wt% of AgNO_3_. Curves A and B show the absorbance results in two different regions of the sample: irradiated and not irradiated with the fs laser. After irradiation with the fs laser, the absorption spectrum revealed an increase in absorbance for wavelengths shorter than 550 nm (curve B in Figure 3a), which is related to the formation of color centers and photoreduction of Ag^+^ ions [24,25]. During fs laser interaction with glass, free electrons are generated due to nonlinear optical interactions, such as photoionization and avalanche ionization [21]. Trapping of the generated electrons can occur (forming color centers) or interact with ions in the glassy matrix, leading to the photoreduction of Ag^+^, which is relevant for the nucleation of NPs [24,25]. During our fs laser processing, the nucleation of the NPs occurs due to the continuous heating of the focal region that promotes atomic mobility, leading to the enhancement of NP growth [25].

Figure 3b presents the PL results (for the bulk samples with and without Ag NPs) at 900, 1064, and 1340 nm, corresponding to the respective transitions: ^4^F_3/2_ → ^4^I_9/2_, ^4^F_3/2_ → ^4^I_11/2_, ^4^F_3/2_ → ^4^I_13/2_. We noticed an improvement of ~47% when compared to the sample without Ag NPs. This improvement was attributed to the increased local field around the Ag NPs, which enhances the density of excited Nd^3+^ ions [9,10,11]. 

Transmission electron microscopy shown in the inset of Figure 3a shows that most of the nucleated NPs were not perfectly spherical in shape. The field increase was considerably larger for non-spherical NPs than for a spherical particle of comparable size [11,26,27] because of the “lightning rod effect” (the accumulation of charges in regions of smaller radius of curvature [28]), and, therefore, non-spherical NPs (size in the range of 8 nm–36 nm) and irregularly shaped aggregates contributed to obtain a large increase in infrared PL. Larger particles and aggregates presented even higher dipole, quadrupole, and multipolar resonances whose excitation promoted greater local fields external to the particles when compared with those produced by isolated NPs [11,29]. 

### 3.2. Raman Results

**Figure 4:**
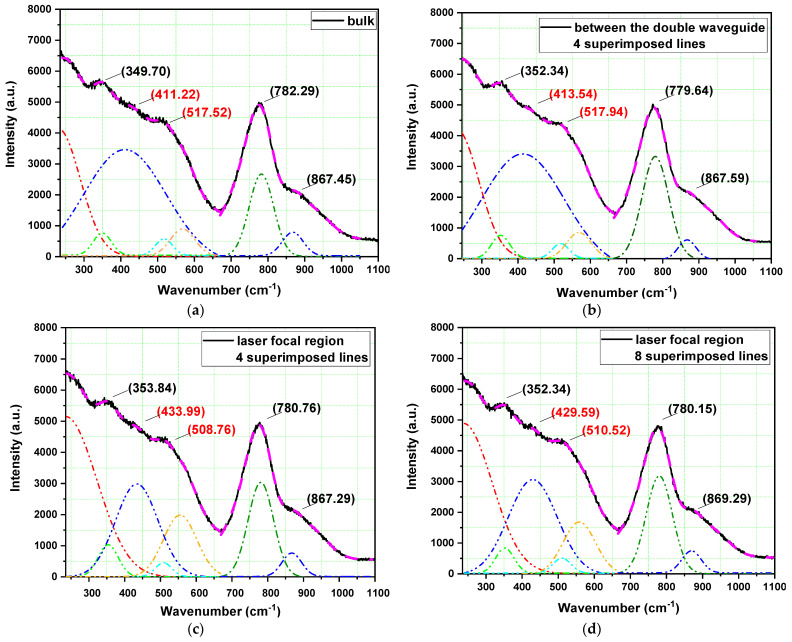

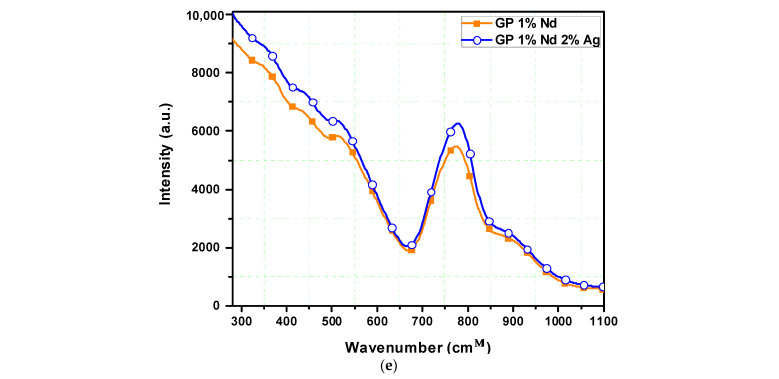
Raman results of Nd^3+^-doped Ge_2_O-PbO glass with Ag NPs in (**a**) the bulk area and (**b**) between the double waveguide written with 4 superimposed lines; Raman results inside the fs written lines of the double waveguide with (**c**) 4 superimposed lines and (**d**) 8 superimposed lines; and (**e**) Raman results in the bulk area of Nd^3+^-doped Ge_2_O-PbO glass with (open circles) and without (square) Ag NPs.

Figure 4a presents Raman results for the bulk glass, and Figure 4b presents those obtained between the two written lines (four superimposed lines), leading us to conclude that no appreciable structural change took place in the region between the two lines (where guidance occurs) by the fs laser processing technique, as previously reported [6,7,8].

When we compared the Raman results of Figure 4a (bulk) and Figure 4b (between the double waveguide) with those of Figure 4c,d (in the laser focal region with four and eight superimposed lines, respectively), we noticed major changes for the peak at 411.2 cm^−1^ (bulk) that displaces to 433.9 cm^−1^ and 429.6 cm^−1^, suggesting modifications in the symmetric stretching vibrations of the Ge-O-Ge bonds, attributed to the irradiation by the fs laser. The Raman shift changed more in this case when compared to Nd^3+^-doped GeO_2_-PbO glass without Ag NPs [8], and it also demonstrated the influence of Ag NPs during the processing of the waveguides. The small displacement of the peak at 517.5 cm^−1^ (bulk) to 508.1 and 510.5 cm^−1^ for written regions with four and eight superimposed lines, respectively, corresponded to a slight change of the symmetric stretching vibrations along the Ge-O-Ge chain [6,30] induced by the waveguides produced by fs laser. The peaks at 782.3 cm^−1^ and at 867.4 cm^−1^ (bulk) corresponded to Ge-O^−^ and Ge-O-Ge symmetric stretching vibrations in the GeO_4_ tetrahedral units [6,30] and asymmetric stretching vibrations of Ge-O-Ge bonds, respectively; a slight change of these peaks was noticed in the laser focal region, indicating little influence of the laser irradiation process too. It is important to highlight that a Raman shift due to laser-induced modification of the structure was also reported for other glass [31,32].

Figure 4e shows the Raman results for the bulk samples with and without Ag NPs. We noticed for all the peaks an intensity enhancement of about 10% in the presence of Ag NPs attributed to the surface plasmon resonance contribution, which enlarged the local electric field in the interface between the Ag-NPs and the dielectric medium, the so-called surface-enhanced Raman scattering (SERS) effect [33,34].

### 3.3. Passive Characterization Results

**Figure 5:**
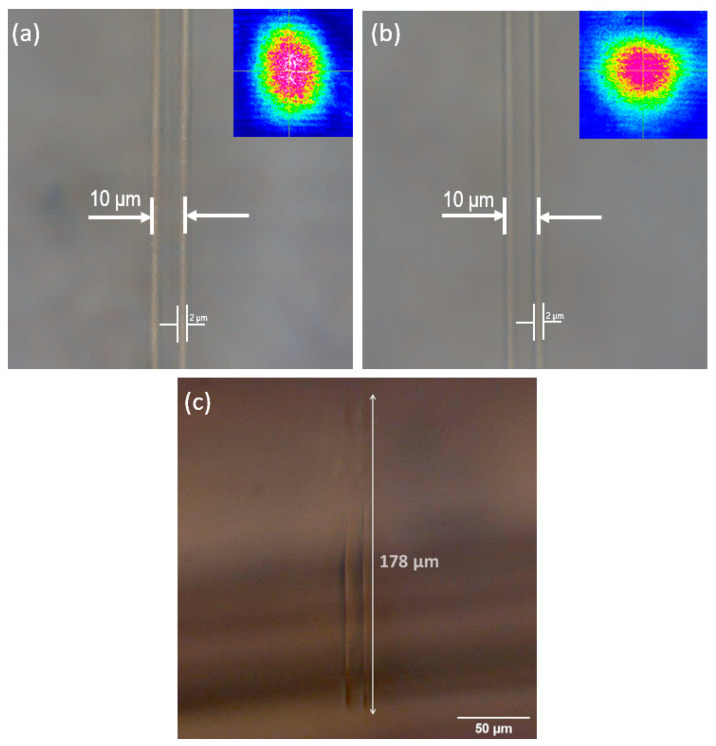
Top view microscope image of the double line waveguide in Nd^3+^-doped GeO_2_-PbO glass with Ag NPs with (**a**) 4 and (**b**) 8 superimposed lines; the near field beam profile (at 632 nm) is presented in the inset for both cases. (**c**) View from the front facet of the 4 superimposed lines showing an apparent depth of focus of 178 µm.

Top images obtained by optical microscopy of dual waveguides written on Nd^3+^-doped GeO_2_-PbO glass with four and eight superimposed lines are presented in Figure 5a,b, respectively; the distance between both lines was 10 μm, and each written line had an apparent (visual) width of 2 μm. Mode images at 632 nm (for waveguides written with four and eight superimposed lines) obtained with the experimental setup of Figure 2a are presented in the insets, evidencing the presence of confined beams. Figure 5c shows a view taken from the beam exit facet of the focal region of both parallel written lines (the femtosecond laser is incident from the top, and the photo shows a cross section of the double waveguide) showing a highly elongated region (the three-dimensional wall mentioned in Section 2.1) of refractive index change of 178 μm extension along the laser’s focus. The low numerical aperture chosen (0.23) decreased the autofocusing effects and enhanced the length of the cross section (seen in Figure 5c) of the refractive index modification that produced the superimposed lines presented in Figure 5a,b.

The refractive index change at 632 nm was determined by Equation (1), and the index change created was negative in the focal region of the written lines. The values for both directions were in the order of 10^−3^ for dual waveguides inscribed with four and eight coincident lines and are reported in Table 2.

**Figure 6:**
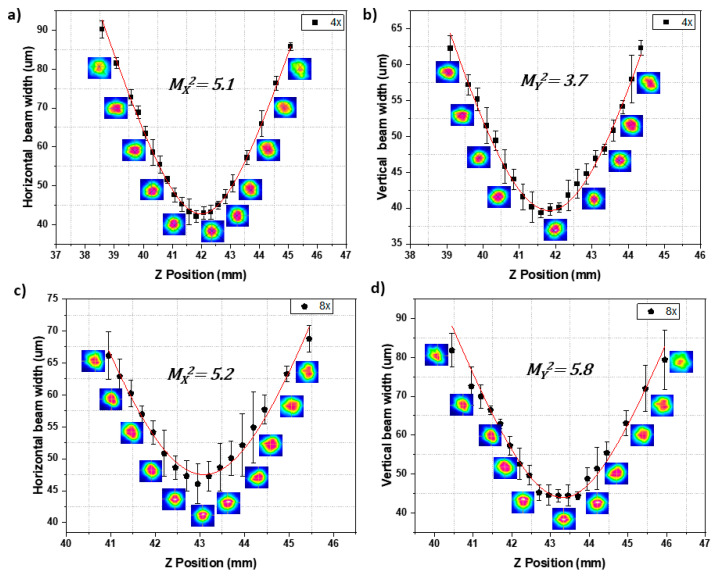
Results of *M_x_*^2^ and *M_y_*^2^ at 632 nm for the different parameters used for the fs laser irradiation for (**a**,**b**) 4 and (**c**,**d**) 8 superimposed lines.

Figure 6 presents the results of *M_x_*^2^ and *M_y_*^2^ at 632 nm for the different fs laser irradiation conditions obtained with the experimental setup shown in Figure 2b. *M_x_*^2^ and *M_y_*^2^ at 1064 nm were calculated with Equation (2). More similar values in the horizontal and vertical directions were achieved at 632 and 1064 nm for eight superimposed lines, showing better x,y-symmetrical guiding than for four superimposed lines. These results are also reported in Table 2 and are compared with the values of the dual waveguides inscribed with four and eight overlapping lines in GeO_2_-PbO glass doped with Nd^3+^ without Ag NPs. From Table 2, it is possible to notice that in the presence of the AgNO_3_ dopant, the M^2^ values improved significantly for the double waveguides with four and eight superimposed lines, probably due to the nucleation improvement of Ag NPs.

It has been reported that the presence of Ag NPs in silicate glass leads to an increase in the refractive index change by 4.6%, favoring the application of direct writing by fs laser for the construction of waveguides [35]. In this work, the refractive index change remained of the same order of magnitude, as can be seen in Table 2.

The propagation losses (α) were determined as explained in Section 3, using Equation (3), and the values obtained at 1064 nm were 1.89 and 1.30 dB/cm for four and eight overlapping lines, respectively. Low propagation losses were obtained with and without Ag NPs (with a small increase in samples with Ag NPs).

In Table 2, *G_INT_* represents the internal gain that will be discussed in the next section.

### 3.4. Active Characterization Results

**Figure 7:**
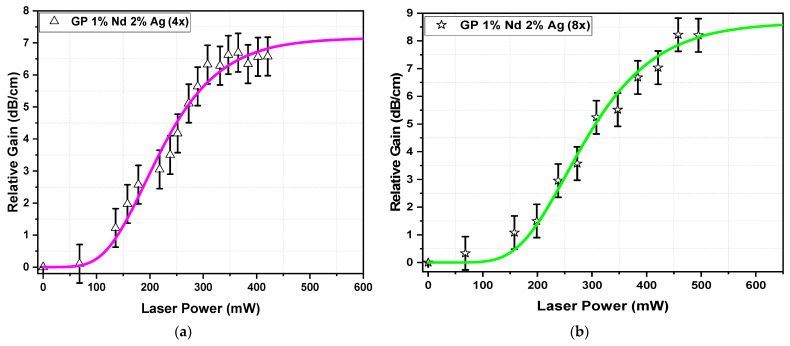
Relative gain (signal enhancement) at 1064 nm as a function of pump power at 808 nm, for 400 nW of input signal power, for the double waveguides written in Nd^3+^-doped GeO_2_-PbO with Ag NPs with (**a**) 4 and (**b**) 8 superimposed lines.

Figure 7a,b show the results of the relative gain at 1064 nm as a function of the pump power (808 nm) for 400 nW of input signal power; the relative gain was calculated using the experimental procedure and Equation (4) presented at the end of Section 2.2. The violet and green curves are fittings (Gompertz function) performed on the experimental points, representing the relative gain. Relative gains of 7.0 and 8.5 dB/cm were obtained for four and eight superimposed lines, respectively, for 600 mW nm pump power at 800 nm. We highlight the Nd^3+^-doped GeO_2_-PbO glass with Ag NPs with waveguides written with eight superimposed lines that exhibited the best condition for optical amplifier applications at 1064 nm. For the sample without Ag NPs, the waveguides with eight superimposed lines also presented the best optical amplification performance, as was already reported [8].

Considering the results obtained for the propagation loss (α), the internal gain could be calculated by *G_INT_ = G_R_ − α* [17]. Then, results of positive internal gain of 5.11 and 7.2 dB/cm were found for four and eight superimposed lines, respectively, demonstrating increases of ~40% and ~30% with respect to the samples without Ag NPs [8], as can be seen in Table 2. This growth was correlated to the PL results presented in Figure 3b, attributed to the intense local field that appeared in the vicinity of Ag NPs that increased the density of excited Nd^3+^ ions.

## 4. Conclusions

The effects of Ag NPs on the optical properties of Nd^3+^-doped GeO_2_-PbO glass with double line waveguides written by a Ti:sapphire are presented. The following parameters were used for the laser irradiation: speed and pulse energy of 0.5 mm/s and 30 µJ, respectively; operating wavelength of 800 nm; and pulse duration of 30 fs at 10 kHz repetition rate. 

This double line architecture formed by a pair of parallel lines, written with spacing of 10 µm, where each written line is formed by four or eight separately written lines that are coincident, was developed in order to overcome the problem of heating, as neodymium absorption at 800 nm is in resonance with the fs laser’s emitting wavelength, thereby easily causing fracture of the glass. This platform was successfully used for Nd^3+^-doped GeO_2_-PbO glass without metallic NPs that are included for reference in the present study. The negative refractive index changes at 632 nm were 10^−3^ in both horizontal and vertical directions. More similar values for the beam quality factors *M_x_*^2^ and *M_y_*^2^ were found at 632 and 1064 nm for eight superimposed lines, showing better x,y-symmetrical guiding with respect to four superimposed lines. A photoluminescence increase of ~47% at 1064 nm with respect to the sample without Ag NPs was observed due to the intense local field that appeared in the vicinity of Ag NPs, which enhanced the density of excited Nd^3+^ ions. The relative gain at the signal wavelength (1064 nm) reached 7.0 and 8.5 dB/cm for four and eight superimposed lines, respectively, for excitation at 808 nm pump power; saturation around 600 mW was observed for both cases. Propagation losses of 1.89 and 1.30 dB/cm were obtained for four and eight superimposed lines, allowing for positive internal gain values of 7.0 and 8.5 dB/cm, respectively. When compared to the samples without Ag NPs, these values represented an increase of ~40% and 30%, which was also attributed to the plasmonic effects of Ag NPs. The present work brings a contribution to the state of the art, as it is the first time, as far as we are concerned, that the influence of Ag NPs on the optical performance of a waveguide irradiated by fs laser writing was studied. Moreover, there is a gap in the literature regarding results of relative and internal gains for waveguides processed by fs laser writing, as normally the passive characterization using undoped waveguides produced by fs laser writing is reported. This present platform with the addition of metallic NPs can be used for photonic applications in different areas of the electromagnetic spectrum and can be extended to different hosts and metallic NPs; moreover, it represents a promising strategy for photonic devices such as the manufacture of lasers and integrated amplifiers.

## Figures and Tables

**Table 1:** Parameters used in the waveguide processing.

Parameters	Values
Writing speed (mm/s)	0.5
Wavelength (nm)	800
Repetition rate (kHz)	10
Pulse energy (µJ)	30
Temporal pulse length (fs)	30
Focal point	0.7 mm beneath the surface
Number of collinear, superimposed lines	4 and 8

**Table 2:** Results of *M_x_*^2^, *M_y_*^2^, Δ*n_x_*, Δ*n_y_*, propagation losses and *G_INT_* (pulse energy of 30 µJ and processing speed of 0.5 mm/s).

	GeO_2_-PbO 1% Nd [8]		GeO_2_-PbO 1% Nd 2% Ag	
Parameters	4 Superimposed Lines	8 Superimposed Lines	4 Superimposed Lines	8 Superimposed Lines
*M_x_*^2^ (at 632 nm)	16.7	16.6	5.12	5.22
*M_y_*^2^ (at 632 nm)	14.2	15.6	3.66	5.82
*M_x_*^2^ (at 1064 nm)	9.9	9.9	3.0	3.1
*M_y_*^2^ (at 1064 nm)	8.4	9.2	2.2	3.5
Δ*n_x_*	−5.7 × 10^−3^	−7.3 × 10^−3^	−5.5 × 10^−3^	−8.1 × 10^−3^
Δ*n_y_*	−5.4 × 10^−3^	−5.3 × 10^−3^	−4.3 × 10^−3^	−4.2 × 10^−3^
Propagation losses (dB/cm)	0.89	0.44	1.89	1.30
*G_INT_* (dB/cm)	3.61	5.56	5.11	7.12

## Data Availability

The data presented in this study are available upon request from the corresponding author.
